# Timing Effects of Heat-Stress on Plant Ecophysiological Characteristics and Growth

**DOI:** 10.3389/fpls.2016.01629

**Published:** 2016-11-02

**Authors:** Dan Wang, Scott A. Heckathorn, Kumar Mainali, Rajan Tripathee

**Affiliations:** ^1^International Center for Ecology, Meteorology and Environment, School of Applied Meteorology, Nanjing University of Information Science and TechnologyNanjing, China; ^2^Department of Environmental Sciences, University of Toledo, ToledoOH, USA; ^3^Department of Biology, University of Maryland, College ParkMD, USA; ^4^Department of Biological Sciences, Rutgers University, NewarkNJ, USA

**Keywords:** global climate change, photosynthesis, aboveground productivity, *Solidago canadensis*, *Andropogon gerardii*

## Abstract

Heat-waves with higher intensity and frequency and longer durations are expected in the future due to global warming, which could have dramatic impacts in agriculture, economy and ecology. This field study examined how plant responded to heat-stress (HS) treatment at different timing in naturally occurring vegetation. HS treatment (5 days at 40.5°C) were applied to 12 1 m^2^ plots in restored prairie vegetation dominated by a warm-season C_4_ grass, *Andropogon gerardii*, and a warm-season C_3_ forb, *Solidago canadensis*, at different growing stages. During and after each heat stress (HS) treatment, temperature were monitored for air, canopy, and soil; net CO_2_ assimilation (*A*_net_), quantum yield of photosystem II (Φ_PSII_), stomatal conductance (*g*_s_), and internal CO_2_ level (*C*_i_), specific leaf area (SLA), and chlorophyll content of the dominant species were measured. One week after the last HS treatment, all plots were harvested and the biomass of above-ground tissue and flower weight of the two dominant species were determined. HS decreased physiological performance and growth for both species, with *S. canadensis* being affected more than *A. gerardii*, indicated by negative HS effect on both physiological and growth responses for *S. canadensis*. There were significant timing effect of HS on the two species, with greater reductions in the net photosynthetic rate and productivity occurred when HS was applied at later-growing season. The reduction in aboveground productivity in *S. canadensis* but not *A. gerardii* could have important implications for plant community structure by increasing the competitive advantage of *A. gerardii* in this grassland. The present experiment showed that HS, though ephemeral, may promote long-term effects on plant community structure, vegetation dynamics, biodiversity, and ecosystem functioning of terrestrial biomes when more frequent and severe HS occur in the future.

## Introduction

The increased concentration of CO_2_ and other greenhouse gasses in atmosphere is causing a future climate with higher temperatures and dramatic changes in rainfall patterns (IPCC, 2013). In addition to rising mean annual temperatures, the frequency, duration, and severity of periods with exceptionally high temperatures are also increasing (Easterling et al., 2000; Tripathi et al., 2016). HS events with a trend of high frequency and extremity have already been reported in different parts of the world (Henderson and Muller, 1997; Gaffen and Ross, 1998; Yan, 2002). Thus, plants in the future will be exposed to both higher mean temperatures, and likely more extreme HS. The World Meteorological Organization (WMO) defines HS events as episodes of 5 or more continuous days with air temperatures over 5°C above daily maximum temperatures (Frich et al., 2002). During extreme climate events the acclimatory capacities of an organism are substantially exceeded (Gutschick and BassiriRad, 2003) and the impact of extreme climate events can be significantly greater than those associated with mean temperature increases (Karl et al., 1997). Combining the climatological and biological definitions, Smith (2011) stated that an extreme climate event is an episode in which a statistically rare climatic period could cause community responses, with loss of key species, invasion by novel species, and alteration ecosystem structure and/or function outside the bounds of normal variability. Therefore, extreme climate events, in spite of their ephemeral nature, can potentially cause shifts in the structure of plant communities (Smith, 2011) and greatly impact ecosystem productivity (Ciais et al., 2005) and biodiversity (Thomas et al., 2004). It is, however, difficult to determine whether the ecological response is explicitly attributable to an extreme climate event, since it may not be extreme enough to cause ecological consequences (Niu et al., 2014).

Accordingly, research has started to not only focus on the impact of the trend of gradual increases in mean temperatures but also on the effects of increasing extreme HS events (Brown et al., 2004; Jentsch et al., 2011; Sentis et al., 2013). However, due to the difficulties in conducting experiments and simulating extreme heat events under field conditions, they are most frequently conducted under controlled conditions in the laboratory (Wang et al., 2008a, 2012). Therefore, the effects of extreme heat events on the vegetation structure and dynamics remained less well understood than effects of climate warming and atmospheric CO_2_ enrichment, especially on crops photosynthesis, respiration, and growth (Long et al., 2004; Ainsworth et al., 2008). To date, only a few experiments with HS treatment have been conducted in plant communities, and these studies focused on recolonization, competition, invasion, and the role of species richness during extreme events in community processes. HS manipulations were conducted mostly on grassland (White et al., 2001; Van Peer et al., 2004) or arctic species (Marchand et al., 2005, 2006). In this study, we will apply short-term HS treatment in a restored prairie and concentrate on the ecophysiological and growth responses of two dominant warm-season tall-grass prairie species with contrasting photosynthetic pathways (a C_4_ grass and a C_3_ forb) to HS.

The negative effects of HS on plants growth and crop yield mainly was caused through its negative effects on photosynthetic process, which is among the most thermosensitive aspects of plant functions (Wang et al., 2008a). Due to inter-annual variations in the timing and duration of hot days, HS events may affect plant physiological processes and community structure differently. Hence, the response of plants photosynthetic activity to HS will depend on the season and growing stage when HS events occur (Xu and Baldocchi, 2003; Yu et al., 2003; Richardson et al., 2010). Although the research of the effects of the timing of the extreme events is urgently needed, there is still a lack of studies in this regard (Jentsch et al., 2011; Smith, 2011). We urgently need to advance research on the effect of the timing of extreme events and their consequences by collecting evidence from experimental studies in natural field conditions. Therefore, this study will simulate HS events in the key phenological stages of the dominant species in an old prairie to investigate how variation in the timing of HS events during the growing season influences physiological processes and growth of individual species and community dynamics.

The optimal temperature for photosynthesis is typically higher for C_4_ species than for C_3_ species because C_4_ species usually have higher water use efficiency and lower photorespiration due to its CO_2_-accumulating mechanisms in the leaf (Sage and Monson, 1999). This may contribute to greater tolerance to HS for C_4_ species than co-occurring C_3_ species (Coleman and Bazzaz, 1992; Ehleringer et al., 1997; Wang et al., 2008b). Differential sensitivities to HS among different species may lead to divergent responses in these dominant species, particularly if the stress exceeds species-specific physiological thresholds (Gutschick and BassiriRad, 2003). In natural systems, the significance of climate warming for C_4_ vegetation can depend less on the mean increase in global temperature and more on the spatial and temporal variation of the temperature increase (Sage and Kubien, 2003). In New Zealand, for example, episodic heat events inhibit C_3_ plants more than C_4_ grasses, and as a result, facilitate C_4_ grass invasion of C_3_-dominated grasslands (White et al., 2000, 2001). However, whether the timing of HS events impacts differently on C_3_ vs. C_4_ species remains to be determined and the differences in the responses to HS applied at different growing stages will have a bearing on the relative impact of global environmental change on the abundance, productivity and distribution of C_3_ and C_4_ species and therefore community structure.

To examine the influence of HS on plants ecophysiological and growth response in naturally occurring mixed C_3_–C_4_ vegetation, we conducted a field study and aimed with the following two major objectives: (1) to determine how HS affects the ecophysiological and morphological characteristics of a C_4_ and C_3_ species which co-dominate a restored prairie community; (2) to determine the effect of timing of HS on each species growth and physiology. Our specific hypotheses were as follows: (1) HS will have a less pronounced negative effect on the C_4_ than the C_3_ species; (2) differences in the responses to HS applied at different growing stage (HS timing effect) will lead to differences in plants ecophysiological responses and growth.

## Materials and Methods

### Field Site and Experimental Treatments

The experiment site was located within a restored prairie vegetation at the University of Toledo’s Stranahan Arboretum (Toledo, OH, USA), within the oak-savannah glacial-sand ecosystem referred to as “Oak Openings” region^1^. *Andropogon gerardii* (big bluestem), a warm-season C_4_ perennial grass, and *Solidago canadensis* (goldenrod), a warm-season C_3_ perennial herbaceous dicot, together account for almost 95% plant canopy cover and the majority of total aboveground productivity in this ecosystem. Top-vented 1 m^3^-chambers made with transparent plastic attached to a wooden frame was used to simulate HS treatment. Heat treatment was applied *in situ* from June 21 to June 25, July 22 to July 26, and August 28 to September 1 in 2007 (as in Wang et al., 2008b). There was no obvious drought situation before each heat treatment. For each heat treatment, eight 1 m × 1 m plots were selected randomly for use; four were untreated controls and four were heated to 39–41°C daytime temperature. A portable electric heater (Heat Runner model 33551, 1500 W), suspended near a corner of the chamber, was used to increase and regulate chamber temperature, and a fan was used to distribute warm air inside the chamber. Spatial variation in temperature within chambers was found to be minimal. The temperature in the central chamber was 0.5 ± 0.3 (standard deviation)°C higher than the edge of the chamber. Plants were not watered during the heat treatment. This experimental design did not allow for determination of chamber effects on plants (increased humidity, decreased wind, and slightly decreased light levels), but such effects would only serve to minimize the negative effects of HS, and make detection of heat effects more difficult. Air temperature was monitored continuously, with either a temperature probe and data logger (HOBO8, Onset Computer Corp, Bourne, MA, USA) or a fine-wire thermocouple and data logger (LI-1000, LiCOR, Lincoln, NE, USA). Leaf temperature was measured with an IR thermometer (cross-checked against the probes above). Soil temperature (10 cm) during midday and at the end of the HS treatment was monitored with a temperature probe and a thermometer (Wang et al., 2008b; Mainali et al., 2014). Heat-treatments in this study were chosen to represent those HS events encountered by vegetation in the Toledo region (northwest Ohio, USA) during summer months. On average, there is about 10 days of HS in July and August during which day time maximal temperatures are higher than 32°C in Toledo. The recorded daytime maximal temperatures for June, July, and August were 40, 41, and 39°C, respectively, and the mean daytime maximal temperatures for June, July, and August were 28, 30, and 29°C, respectively^2^. Therefore, the target HS treatment temperature was set at 40, 41, and 39°C for June, July, and August, respectively, in this experiment.

### Gas Exchange and Leaf Trait Measurements

Photosynthetic measurements were conducted during and after each HS treatment in order to determine the timing effect of HS on foliar gas exchange. During and after each heat treatment, one fully expanded leaves were chosen randomly from each plot and net photosynthetic rate, stomatal conductance to water vapor, and internal CO_2_ level were measured daily with a portable infrared gas analyzer (LI-COR 6400LCF; LI-COR, Lincoln, NE, USA). During measurements, CO_2_ concentration of 380 μmol mol^-1^, leaf temperature of 25°C, photosynthetic photon flux (PPFD) of 1500 μmol m^-2^ s^-1^ and airflow through the chamber of 250 μmol s^-1^ were set in the leaf chamber. Net photosynthetic rate (*A*_net_) was taken as the rate of photosynthesis at a PPFD of 1500 μmol m^-2^ s^-1^. The parameters including stomatal conductance (*g*_s_) and intercellular CO_2_ concentration (*C*_i_) were recorded during the photosynthetic measurement. Intrinsic water use efficiency (_i_WUE) was calculated as the ratio of net photosynthetic rate to stomatal conductance. Quantum yield of PSII electron transport (Φ_PSII_) was measured with a pulse-amplitude-modulated (PAM) fluorometer with a saturating pulse of 3000 μmol photons m^-2^ s^-1^ (Model PAM 101/103, Walz, Germany) on ambient light-adapted (∼800 μmol photons m^-2^ s^-1^) plants, as in Wang et al. (2008b). Leaf area index (LAI) was measured once per week, using LAI-2000 (LI-COR Biosciences, Lincoln, NE, USA). After gas-exchange measurements of last heat-stress treatment, ten 0.5 cm^2^ leaf punches from each leaf were taken and oven-dried at 65°C for 2 weeks for measurement of SLA (m^2^ kg^-1^) and LWC (%). An index of the total leaf chlorophyll content was measured using a chlorophyll meter (SPAD-502, Konica Minolta, Japan). Readings were taken along the middle section of the four leaves of one plant and the mean value was used for analysis. The measurements were made on five plants from each treatment before, during and after the HS.

### Biomass and *C, N* Measurements

Four-week after the last HS treatment, 40 cm × 50 cm of each plot was harvested. The clipped plants were sorted into different categories (species, green and senescent leaves, stems and flowers), oven-dried at 65°C for 1 week and weighed.

### Statistical Analysis

All statistics were tested in the R statistical language^3^. The normality of the residuals of all the variables was tested using the Shapiro–Wilk test. Fixed effects of species, heating stress at different time and their interactions on the morphological, biochemical, and physiological parameters were tested by a linear mixed-effects model, using the lme4 package^4^. The measuring time were specified as a random factor to control for their associated intra-class correlation. Linear mixed-effects models also tolerate the necessarily unequal number of responses and unbalanced sample sizes for each treatment. We obtained *p*-values for regression coefficients using the nlme package. For the sake of brevity, we present only the *F* tests from the LMER results here (type III Wald F tests with Kenward–Roger degrees of freedom approximation). A *Post hoc* Tukey HSD tests were made on specific contrasts to examine significant treatment effects among groups (step function in the nlme package, R). End of season measurements of aboveground primary production, flower weight and leaf morphological parameters were analyzed via *t*-tests to account for heat-stress timing effect. All statistical tests were considered significant at *P* ≤ 0.05. Mean values of each variable were expressed with their standard error (SE).

## Results

**FIGURE 1:**
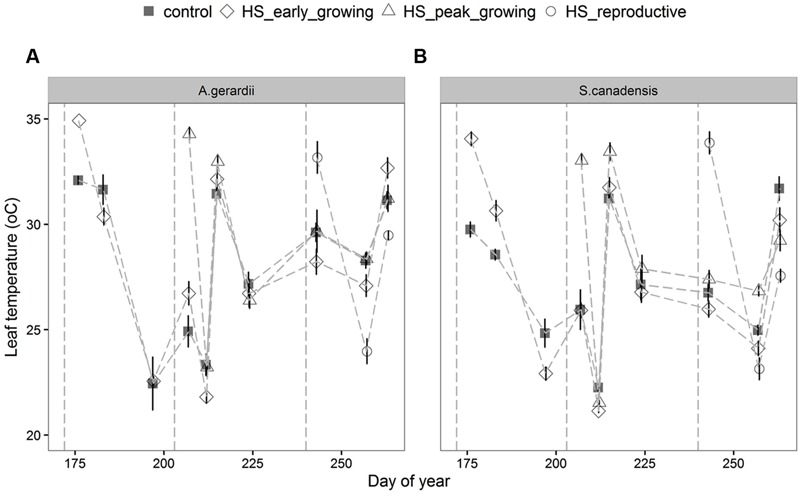
**Effects of HS applied at different growing stage on leaf temperature of **(A)***Andropogon Gerardii* and **(B)***Solidago Canadensis*.** Measurements were taken during and after each heat-stress treatment. Values are means ± 1 SD; *n* = 4.

Air temperature in the heated plots increased on average to 40.5 ± 2.8°C during HS treatment (data not shown, as in Wang et al., 2008b; Mainali et al., 2014). During the 5-days HS treatment, leaf temperature of *A. gerardii* and *S. canadensis* in heated plots was higher than that in control plots, but returned to control levels right after the end of HS (**Figure 1**; **Table 1**). For HS applied during early-, peak-, and reproductive- growing season, leaf temperature reached 34.9, 34.3, and 33.2 for *A. gerardii* and 34.1, 34.1, and 33.8°C for *S. canadensis* in the heated plots, respectively (**Figure 1**).

**Table 1:** Degrees of freedom (numerator, denominator) and *F*-statistics from the linear mixed effect model on the fixed effect of treatment on the morphological, biochemical, and physiological parameters.

Factors	LAI	ANPP	Flower weight	Leaf Temp	Φ_PSII_	*A*_net_	*g*_s_	*C*_i_	*T*_r_	_i_WUE	SLA	LWC	*Chl*
Species			1,70 35.6^∗∗^	1,590 16.3^∗∗^	1,583 526.78^∗∗^	1,274 232.8^∗∗^	1,274 69.4^∗∗^	1,274 738.1^∗∗^	1,274 17.0^∗∗^	1,274 656.1^∗∗^	1,123 73.86^∗∗^	1,123 25.50^∗∗^	1,1526 29.0^∗∗^
Treatment	3,122 10.9^∗∗^	3,16 2.88^∗^	3,70 1.2	3,591 13.5^∗∗^	3,583 15.15^∗∗^	3,278 15.1^∗∗^	3,276 2.6^∗^	3,275 7.9^∗∗^	3,278 0.6	3,278 24.6^∗∗^	3,123 3.22^∗∗^	3,123 11.41^∗∗^	3,1505 24.6^∗∗^
Species ^∗^Treatment			3,70 1.0	3,590 0.1	3,583 1.41	3,274 2.3^∗^	3,274 0.2	3,274 1.6	3,274 0.7	3,274 3.6^∗^	3,123 1.69	3,123 0.39	3,1526 1.6

*^∗^Denotes significance at *P* < 0.05; ^∗∗^Denotes significance at *P*< 0.01. The full name and explanation for the symbols were provided in the abbreviation list.*

**FIGURE 2:**
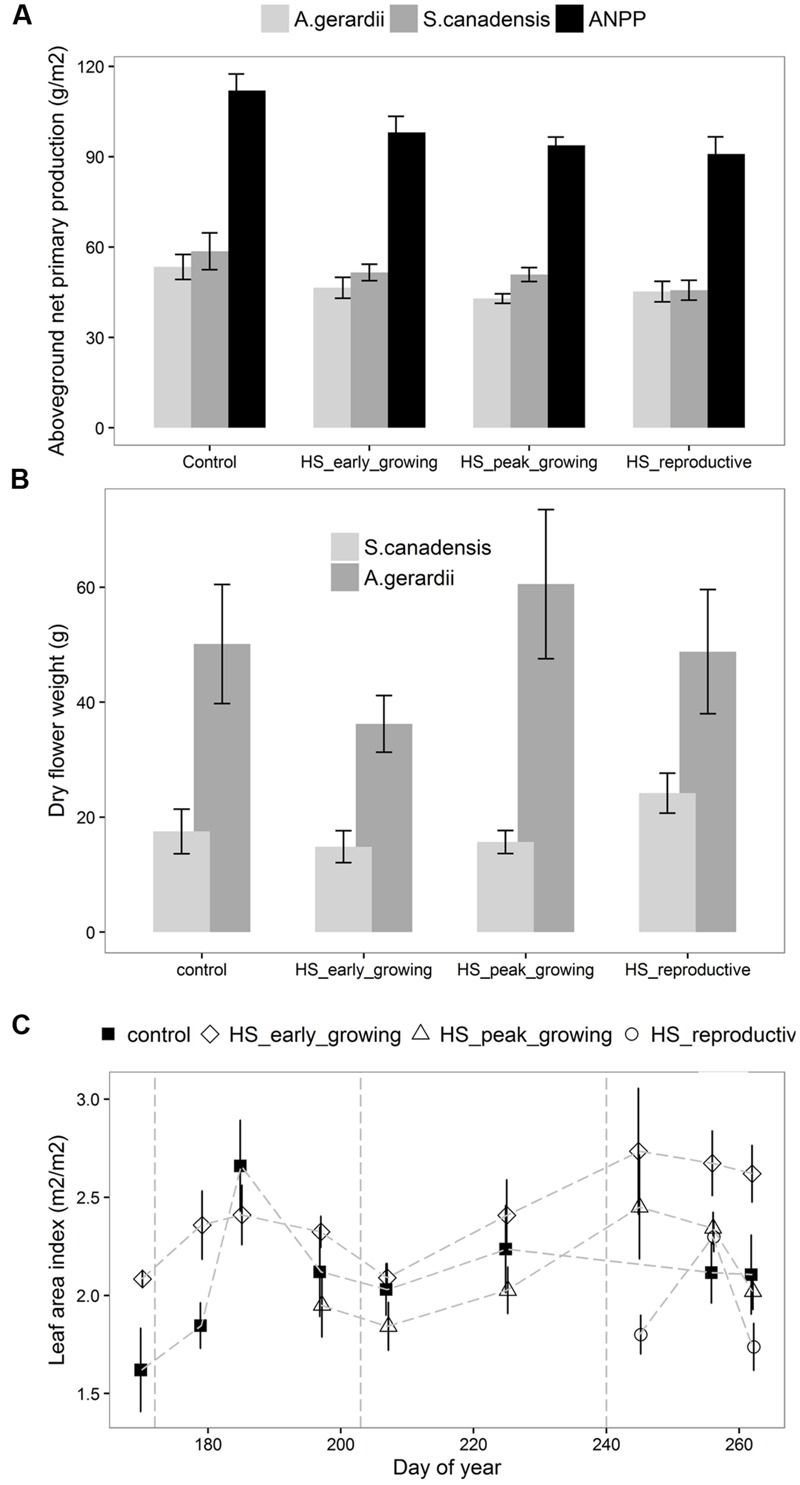
**Effects of HS applied at different growing stage on **(A)** the aboveground net primary productivity, productivity of *A. gerardii* and *S. Canadensis*; **(B)** the flower weight of *A. gerardii* and *S. Canadensis*; **(C)** leaf area index (LAI).** LAI was measured once during and twice after each heat-stress treatment. Values are means ± 1 SD; *n* = 4.

Aboveground net primary production at the end of growing season differed significantly among different treatments (**Figure 2A**). ANPP of the plots heat-stressed at reproductive-growing season was significantly lower than that of the control plots. The productivity of *S. canadensis*, but not *A. gerardii*, was significantly reduced by HS. The flower weight of *S. canadensis* was higher than that of *A. gerardii*, but neither was affected by HS (**Figure 2B**). LAI was significantly lower at the plots heat-stressed at reproductive growing stage than the control plots (**Figure 2C**). LAI was highest at the plots heat-stressed at early-growing season and lowest at the plots heat-stressed at reproductive-growing stage (**Figure 2C**).

**FIGURE 3:**
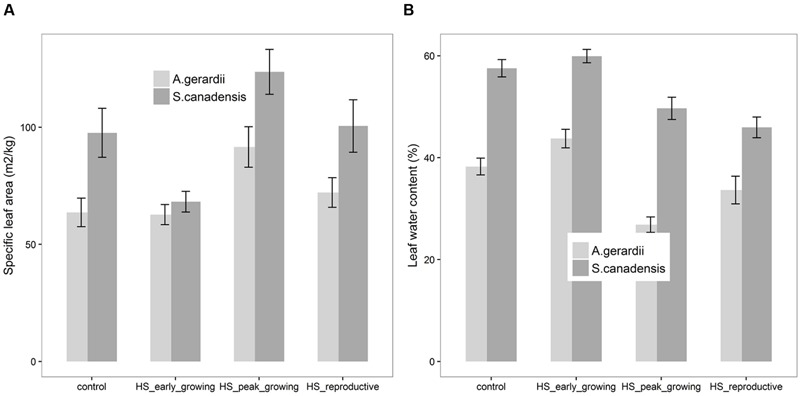
**Effects of HS applied at different growing stage on **(A)** specific leaf area (SLA) and **(B)** relative leaf water content of *A. gerardii* and *S. canadensis*.** Values are means ± 1 SD; *n* = 4.

Specific leaf area of *S. canadensis* was higher than that of *A. gerardii*. Compared with control plots, HS at peak-growing stage significantly increased SLA for both *A. gerardii* and *S. canadensis* (**Figure 3A**). SLA of plants heat-stressed at peak- and reproductive- stages was significantly higher than that heat-stressed at early-growing stages. LWC of *S. canadensis* was higher than that of *A. gerardii*. Compared with control plots, HS at reproductive-growing stages significantly decreased LWC for *S. canadensis*. And for *A. gerardii*, LWC of plants at the plots heat-stressed at peak-growing stage was significantly lower than that at control plots and plots heat-stressed at early-growing stages (**Figure 3B**).

**FIGURE 4:**
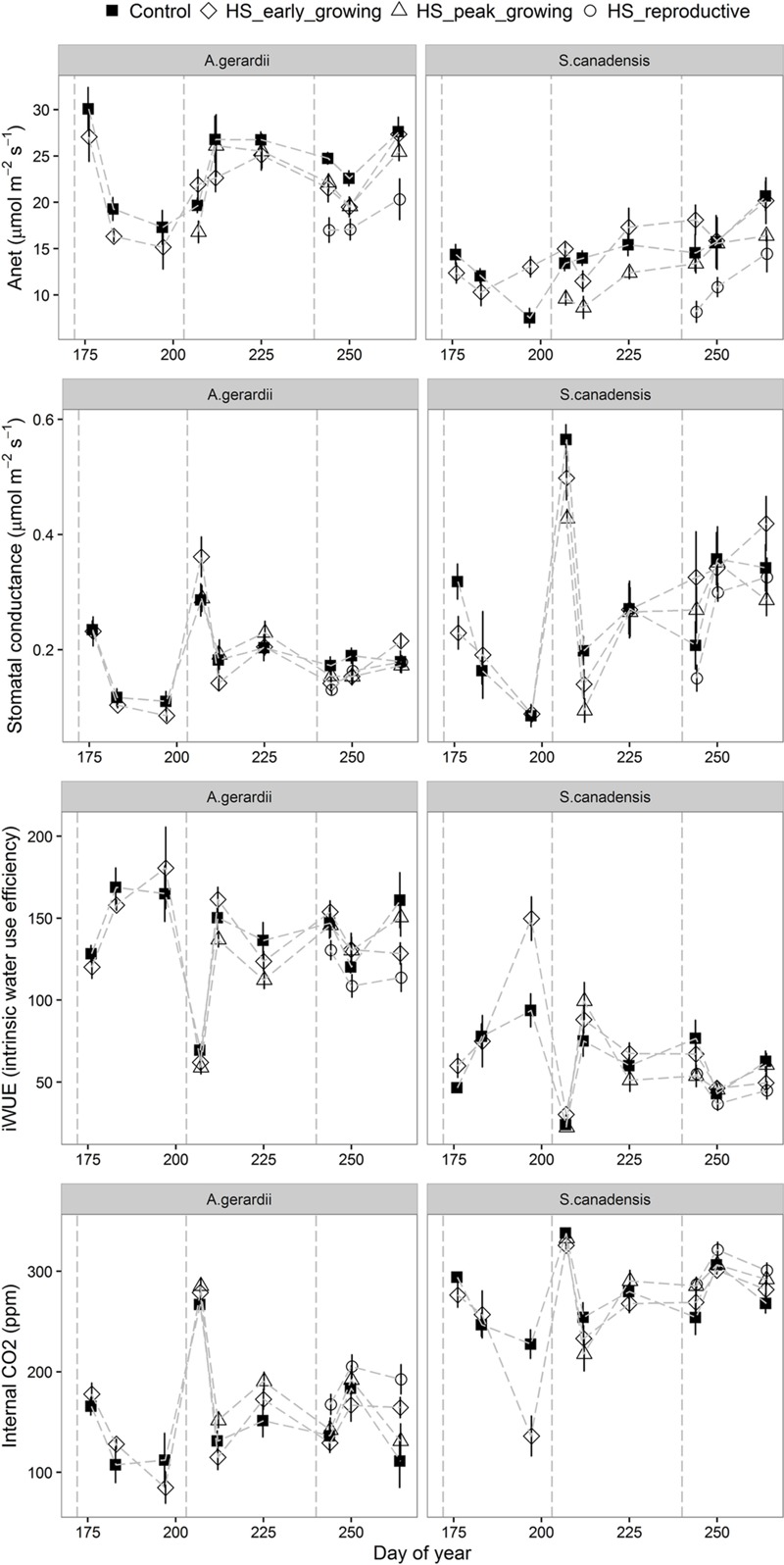
**Effects of HS applied at different growing stage on net photosynthesis (*A*_net_), stomatal conductance (*g*_s_), leaf internal CO_2_ (*C*_i_) and intrinsic water use efficiency of *A. gerardii* and *S. canadensis*.** Measurements were taken once during and twice after each heat-stress treatment. Values are means ± 1 SD; *n* = 4.

Heat stress treatment reduced net CO_2_ assimilation rate and stomatal conductance in heat-stressed plants. *A*_net_ (net photosynthetic rate) was higher for *A. gerardii* than that of *S. canadensis*. *A*_net_ was significantly lower in heated plots than in control plots during HS for *A. gerardii* and *S. canadensis* (statistical results not shown). *A*_net_ remained depressed for at least 1 week after HS in heated plants, relative to unheated plants. Throughout the experimental duration, *A*_net_ was significantly decreased by heat-stress at peak and reproductive growing stages, compared with control plots. *A*_net_ was lowest for the plots heat-stressed at reproductive stage, followed by the plots heat-stressed at peak-growing and early-growing stage (**Figure 4**). Stomatal conductance to water vapor (*g*_s_) varied among different species and treatment. For *A. gerardii* and *S. canadensis, g*_s_ was lower in heated plots. There was also a similar significant timing effect of HS on *g*_s_ as on *A*_net_, with lowest *g*_s_ achieved at plots heat-stressed at reproductive stage (**Figure 4**; **Table 1**). Variation in internal CO_2_ (*C*_i_) was also a function of species and treatment. For *A. gerardii* and *S. canadensis, C*_i_ was higher in heated plots. There was also a significant timing effect of HS on *C*_i_, with highest *C*_i_ achieved at plots heat-stressed at reproductive stage than that of control plots and plots heat-stressed at early- and peak- growing stages (**Figure 4**; **Table 1**). The intrinsic water use efficiency (_i_WUE) was lower for the plants heat-stressed at the reproductive stages than control plants. There was no significant difference between control plots and plots heat-stressed at early- and peak- growing season (**Figure 4**).

**FIGURE 5:**
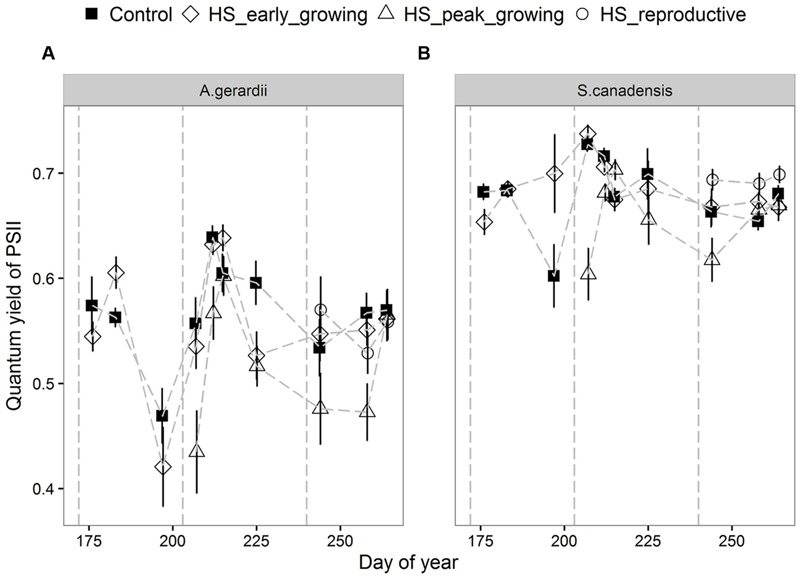
**Effects of HS applied at different growing stage on quantum yield of photosystem II (Φ_PSII_) of **(A)***A. gerardii* and **(B)***S. canadensis*.** Measurements were taken during and after each heat-stress treatment. Values are means ± 1 SD; *n* = 4.

**FIGURE 6:**
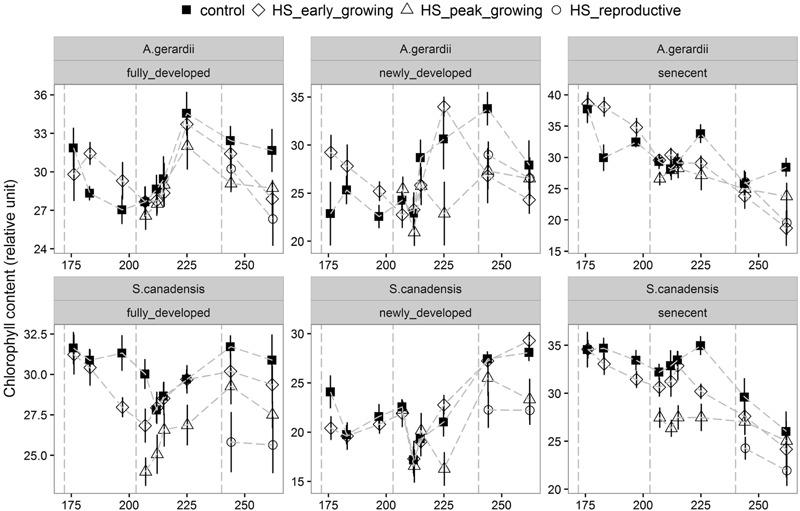
**Effects of HS applied at different growing stage on relative chlorophyll content of the newly develop, fully developed and senescent leaves of *A. gerardii* and *S. canadensis*.** Measurements were taken during and after each heat-stress treatment. Values are means ± 1 SD; *n* = 4.

Quantum yield of PSII electron transport (Φ_PSII_) was higher for *S. canadensis* than for *A. gerardii*. HS at different growing stages played a significant role in affecting Φ_PSII_. HS decreased Φ_PSII_ significantly when it was applied at the peak-growing season, compared with control plots (**Figure 5**). The content of chlorophyll (chlorophyll a + chlorophyll b) was significantly different among different treatments. For both species, chlorophyll content in newly developed leaves was significantly lower than that in the fully developed and senescent leaves (statistical not shown). The chlorophyll content was affected by heat-stress at different growing stages significantly. HS applied at different growing stages all lowered chlorophyll content significantly compared with control plots for both species. Chlorophyll content of the two species heat stressed at peak and reproductive growing stage was lower than that heat-stressed at early growing stages (**Figure 6**).

## Discussion

Extreme climate events have long been acknowledged as a universal phenomenon in recent years and caused great agricultural, economic and ecological consequences (IPCC, 2013). However, in natural field conditions, comprehensive investigations of the effect of HS occurring at different growing stage on plants ecophysiology and growth are still scarce. In this study, we simulated HS events in a tallgrass prairie and collected plant ecophysiological and growth data throughout a growing season. These *in situ* physiological and whole-plant responses of the two dominant species showed different sensitivity to temperature manipulations exposed at different growing stages. Overall, we found that (1) the physiology of both species and growth of *S. canadensis* were affected by HS treatment; (2) the degree of HS effect varied when it applied at different growing stages, with greater negative effect associated with HS applied at later-growing season; (3) the physiology and growth of the two dominant species showed differential sensitivity to HS, with *S. canadensis* being affected more than *A. gerardii*.

Both *A. gerardii* (C_4_) and *S. canadensis* (C_3_) experienced decreased *A*_net_ and intrinsic water use efficiency during HS. The decreases in *A*_net_ were still evident 1 week after heat treatment ended and the recovery to the control level took at least 1 week, which indicates that under moderate HS conditions (most commonly reported at temperatures between 35 and 40°C), photosynthesis can be reversibly reduced (Sharkey and Zhang, 2010; Huve et al., 2011). The direct effects of HS could have led to thermal damage to the photosynthetic machinery. We detected differences in *A*_net_ among the HS treatments persisting after the treatments ended, as well as a negative response in end of season aboveground productivity for *S. canadensis*, so there could have been significant thermal damage to the photosynthetic capacity of the two species. Photosynthesis can be reduced directly through non-stomatal limitations or indirectly through stomatal limitations under HS conditions (Sage et al., 2008; Bussotti et al., 2014). In this study, the decrease in *A*_net_ was associated with either reduced stomatal conductance for *S. canadensis* or down-regulation of quantum yield photosystem II (PSII) for both species (Rennenberg et al., 2006; Sage et al., 2008). Preventing excessive water loss and hydraulic failure through stomatal closure can also limit evaporative cooling and restrict CO_2_ input into the leaf, which is a strategy to save water before further damages happen due to increase in the temperature and/or drought stress (Bauweraerts et al., 2014; Teskey et al., 2014). The restrictions on CO_2_ input to the leaf due to stomatal closure resulted in reduced carbon assimilation (McDowell et al., 2008). In contrast to heat-induced stomatal closure, *A. geraidii* kept stomata relatively open under HS conditions which could enable effective transpirational cooling and prevent leaf from overheating (McDowell et al., 2008). Also, reduced intercellular CO_2_ concentration suggested that CO_2_ concentration also had negative effects on carbon assimilation, as observed previously in other species (Wang et al., 2008a,b, 2012).

When absorbed light are not dissipated efficiently as heat or used in the photosynthetic process, stomatal closure and reduced CO_2_ uptake can lead to the photo-oxidative stress (Demmig-Adams et al., 2012; Foyer et al., 2012). The quantum yield of PSII (Φ_PSII_) measures the proportion of light absorbed by chlorophyll associated with PSII system that is used in photochemistry (Baker and Rosenqvist, 2004). In this study, *S. canadensis* exhibited higher Φ_PSII_ than *A. gerardii* during and after HS, but the decrease of Φ_PSII_ compared to control samples during HS for *A. gerardii* was not significantly different from *S. canadensis* (**Figure 4**; **Table 1**). The significantly decreased Φ_PSII_ suggested that both species engaged flexible heat dissipation in response to HS, presumably because the activation of Rubisco was inhibited at higher temperatures (Feller et al., 1998). The observed reduction of Φ_PSII_ was indicative of acclimation responses or repair processes rather than sustained damages to PSII, because Φ_PSII_ recovered after HS treatment ended. The relative chlorophyll content of the leaves in the two species decreased significantly after HS and most of them did not recover completely, which is more evident in *S. canadensis* and when HS was applied during the later-growing season. HS have also been found to decrease total chlorophyll content significantly in eight Australian wheat varieties when the temperature increased from 28 to 36°C during 6 days (Balouchi, 2010). Efeoglu and Terzioglu (2009) reported that the total chlorophyll content in two wheat cultivars did not change during an 8 h HS treatment of 37°C, but significantly decreased during an 8 h HS treatment of 45°C. The high chlorophyll contents have been associated with heat tolerance in some wheat varieties (Reynolds et al., 1997).

Specific leaf area of both species in this study increased due to HS treatment applied in the peak-growing season. Alterations in leaf structure are an important mode of acclimation in many species (Wright et al., 2005). Higher SLA is beneficial for obtaining higher potential evaporative demand and a more extensive foliar display that captures more light for constant biomass investment (Schuepp, 1993; Niinemets, 1999; Wright et al., 2004). SLA was reported to be higher in higher growth temperatures (Williams and Black, 1993; Loveys et al., 2002), while others reported no systematic temperature-induced change in SLA of five deciduous and evergreen tree species grown at five temperatures (Tjoelker et al., 1999). The impact of temperature on SLA therefore depends on which species is being investigated and the temperature regimes at which the plants are grown and treated.

The optimal temperature for photosynthesis ranges between 20 and 35°C for most plant species (Rennenberg et al., 2006; Sage et al., 2008). However, thermotolerance of photosynthesis to HS differs in different species (Berry and Björkman, 1980) and foliage types (Dreyer et al., 2001; Duan et al., 2014). The responses of photosynthesis to HS depends on adaptation strategies to habitat conditions (Knight and Ackerly, 2002; Cunningham and Read, 2006; Weston and Bauerle, 2007; Gunderson et al., 2010) and climate change scenarios such as CO_2_ elevation (Wang et al., 2008a, 2012). However, the species differences were not always found (Ghannoum et al., 2010) or the thermotolerance of species was reported to be unrelated to the temperature at their site of origin (Lin et al., 2013). In contrast with our hypothesis, both species showed reduced *A*_net_ during HS and the sensitivity of *A*_net_ of the two species responding to the HS did not vary significantly (**Figure 5**; **Table 1**). However, the C_3_ species, *S. canadensis*, tended to close stomata in response to HS, leading to reduced transpiration (and therefore reduced transpirational cooling upon HS). In contrast, the C_4_ species, *A. gerardii*, tended to keep stomata relatively open and maintained high transpiration rates which would limit negative temperature effects on the foliage.

The response of plants to HS was variable depending on the season or life stage during which the HS event happened. Plants were reported to be more susceptible to HS during later reproductive developmental stages (Cross et al., 2003). Early-growing or peak-growing season HS had neutral effects on plants growth for both species. In contrast, later-growing season HS significantly decreased the productivity of *S. canadensis*. Mid- or late- summer heat event was associated with strong physiological stress (De Boeck et al., 2011). In this study, ANPP and LAI of the plots heat-stressed at reproductive season was significantly lower than that of the control plots and the reduction was mostly caused by the negative HS effect on *S. canadensis*. The reduction in ANPP was mostly due to the experimental treatment, as the ratio between the two species in each treatment was not significantly different (**Figure 2A**). Consistently, the physiological performance of *A*_net_, *g*_s_, and Φ_PSII_ were all decreased more during HS applied at peak- or reproductive- growing stages.

Notably, in this study, the flower weight of the two species was not affected by HS (**Figure 2B**), which is contrary to what have reported that heat-stressed plants decreased flower production and produced later flowers on existing inflorescences (Sato et al., 2000; Cross et al., 2003). HS applied at different growing stage had no effect on mortality for the two species (data not shown). *S. canadensis* had similar mortality rate, while *A. gerardii* showed no mortality among different treatments. Andrello et al. (2012) reported increased mortality of juvenile plants in the endangered *Eryngium alpinum* L. during HS, while adult plants were less affected. Similarly, mortality of the Tenerife endemic *Helianthemum juliae* seedling reached nearly 100% in years of extreme drought (Marrero-Gómez et al., 2007).

## Conclusion

Understanding the responses of dominant species to climate extremes is essential to predict future ecosystem dynamics and is particularly important when these species experience similar growing phenology but differ in their sensitivities to the climate factors. In this study, we examined the timing effects of HS on two dominant species in a tallgrass prairie ecosystem. There were two main conclusions drawn from this research. First, the photosynthetic and growth responses of these two species to HS were different, with *S. canadensis* being affected more than *A. gerardii*, indicated by the negative HS effect on both physiological and growth responses for *S. canadensis*; secondly, there were significant timing effect of HS on the two species, with greater reductions in photosynthesis and productivity occurred when HS was applied at later-growing season. The reduction in aboveground productivity in *S. canadensis* but not *A. gerardii* can increase the competitive advantage of *A. gerardii*, which therefore could have dramatic implications for species abundance, distribution and community structure. The present experiment showed that ephemeral HS may promote stochastic successions at the community level (Kreyling et al., 2011) or promote long-term effects on deterministic trajectories at the ecosystem scale (Allen and Breshears, 1998). It is worth pointing out that the negative HS effect in this study may be smaller than likely to occur, as HS treatment applied in this experiment was a single HS event and plants in Northwest Ohio experience multiple HS events per summer. Thus, the negative HS effect could be underestimated in this study and special caution should be paid when to predict long-term heat-stress consequences and differences between C_3_ and C_4_ plants. Furthermore, this study focused on plants ecophysiological processes and only examined short-term plant responses to HS within one generation of perennial plants. The results suggest that long-term effect of HS on plant communities and ecosystems dynamics should be studied more extensively and with longer experimental durations, particularly in combination with other potentially interactive aspects of global environmental change including increases in atmospheric CO_2_ and O_3_ concentration and altered precipitation pattern (Wang et al., 2014a,b).

## Author Contributions

DW and SH came up with the research idea. DW led the experiment and writing. KM and RT assisted conducting the experiment and revising the manuscript.

## Conflict of Interest Statement

The authors declare that the research was conducted in the absence of any commercial or financial relationships that could be construed as a potential conflict of interest.

## Footnotes

1 http://oakopen.org/

2 http://www.ncdc.noaa.gov

3 http://www.r-project.org/

4 http://cran.r-project.org/web/packages/lme4/index.html

## Abbreviations

*A*_net_: net photosynthetic rate (μmol m^-2^ s^-1^)
ANPP: Aboveground net primary production (g)
*g*_s_: stomatal conductance to water vapor (mol m^-2^ s^-1^)
HS: heat stress
LAI: leaf area index (m^2^ m^-2^)
LWC: (leaf water content, %)
SLA: specific leaf area (m^2^ kg^-1^)
*W*_a_: aboveground biomass (g)
*W*_f_: biomass of flowers (g)
_i_WUE: intrinsic water use efficiency (the ratio of *A*_net_ to *g*_s)_
Φ_PSII_: quantum yield of electron transport of photoystem II
